# Seroconversion to HCoV-NL63 in Rhesus Macaques

**DOI:** 10.3390/v1030647

**Published:** 2009-10-30

**Authors:** Ronald Dijkman, H. Lie Mulder, Lynne Rumping, Ilse Kraaijvanger, Martin Deijs, Maarten F. Jebbink, Ernst J. Verschoor, Lia van der Hoek

**Affiliations:** 1 Laboratory of Experimental Virology, Department of Medical Microbiology, Center for Infection and Immunity (CINIMA), Academic Medical Centre (AMC), University of Amsterdam, Meibergdreef 15, 1105 AZ, Amsterdam, The Netherlands; E-Mails: r.dijkman@amc.uva.nl (R.D.); h.l.mulder.1@student.rug.nl (H.L.M.); lynne.rumping@student.uva.nl (L.R.); ilse.kraaijvanger@student.uva.nl (I. K.); m.deijs@amc.uva.nl (M.D.); m.f.jebbink@amc.uva.nl (M.F.J.); 2 Department of Virology, Biomedical Primate Research Centre (BPRC), Lange Kleiweg 139, 2280 GH, Rijswijk, The Netherlands; E-Mail: verschoor@bprc.nl (E.J.V.)

**Keywords:** HCoV-NL63, rhesus macaques

## Abstract

HCoV-NL63 is a recently identified respiratory virus. Its pathogenesis has not been fully unraveled because an animal model is currently lacking. Here we examined whether rhesus macaques encounter HCoV-NL63 infections during life, by examining the levels of antibodies to HCoV-NL63 in time. The animals were followed for 7 up till 19 years, and in three animals we observed a steep rise in antibodies during follow up, indicative of a natural infection with HCoV-NL63.

## 1. Introduction

HCoV-NL63 was identified in 2004 in a child with bronchiolitis in The Netherlands [1], yet worldwide screenings revealed that the virus can be detected throughout the globe in children and adults with respiratory tract infections [2–8]. People acquire their first infection with HCoV-NL63 early in life [9], and during this first infection the chance to develop larynchotracheabronchitis (croup) is high [10]. With increasing age people experience repeated infections (unpublished findings LvdH), and in case of underlying disease an HCoV-NL63 infection can require hospitalization. On the whole we can say that within a few years a vast amount of data on this new virus has become available, except that the pathogenesis of HCoV-NL63 remains unknown. The lacking of an animal model system is the problem. In addition, an animal model system is needed to fulfill the Koch’s postulates and link infection to a disease.

To date, only a limited number of primary or immortalized cells from either human or non-human primate origin support propagation of HCoV-NL63 [1,11–13]. The first HCoV-NL63 isolate that was cultured was propagated on tertiary monkey kidney cells of a cynomolgus monkey (latin: *Macaca fascicularis*) and subsequently passaged to LLC-MK2 cells, a cell line derived from the kidney of a rhesus macaque (latin: *Macaca mulatta*). It is of interest to investigate whether rhesus macaques can be infected by HCoV-NL63. This can either be investigated by direct inoculation of Rhesus Macaque with HCoV-NL63. Alternatively, one can establish whether rhesus macaques that are in close contact to humans have experienced a natural HCoV-NL63 infection by investigating the NL63-directed antibody titers in time.

## 2. Results and Discussion

A sudden rise in coronavirus antibody titers is a strong indication for a coronavirus infection. We have shown this for children who acquire their first HCoV-NL63 infection during their first years of life [9]. At later ages repeated infections with human coronaviruses can occur [14], depending on the levels of protective antibodies. Protective antibodies peak shortly after infection and gradually decrease during the following years, with accompanying susceptibility to a reinfection with time. In the current study we use the kinetics of the antibody response to HCoV-NL63 to determine whether rhesus macaques encounter natural infections with HCoV-NL63, or related coronaviruses. The serological assays were performed with the N-protein of HCoV-NL63 which is an immunodominant protein, although there is some homology with the N-protein of the closest relative of HCoV-NL63, HCoV-229E. To ascertain the most specificity, the C-terminal region (amino acids 215 to 377) of the protein – which has the most difference between NL63-N and 229E-N - was used. In addition competition experiments with homologous and heterologous proteins were included in each experiment to ascertain the specificity of an antibody response.

**Figure 1.**
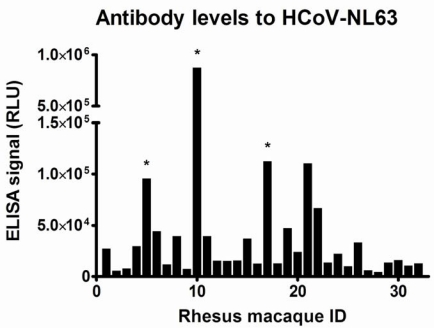
Antibody levels to HCoV-NL63. On the X-axis the Rhesus Macaque ID is indicated. On the Y-axis relative luminescence signal (RLU) in an ELISA detecting antibodies to the C-terminal nucleocapsid protein is shown. Rhesus macaques of which longitudinally collected serum samples were analyzed are indicated with a “*”.

The first indication of infections with HCoV-NL63-like viruses in rhesus macaques was found when we tested 32 serum samples of 32 monkeys (Table 1 and Figure 1). Five samples showed high levels of antibodies to the HCoV-NL63 protein. The same samples did not have elevated levels of antibodies to HCoV-229E, indicative that the response was specific (data not shown). Furthermore, competition experiments with the NL63-protein and the 229E-protein revealed that only the homologous protein could compete with binding to the NL63-protein on the ELISA plate (data not shown), suggesting that the antibodies are directed to an HCoV-NL63-like virus.

**Figure 2.**
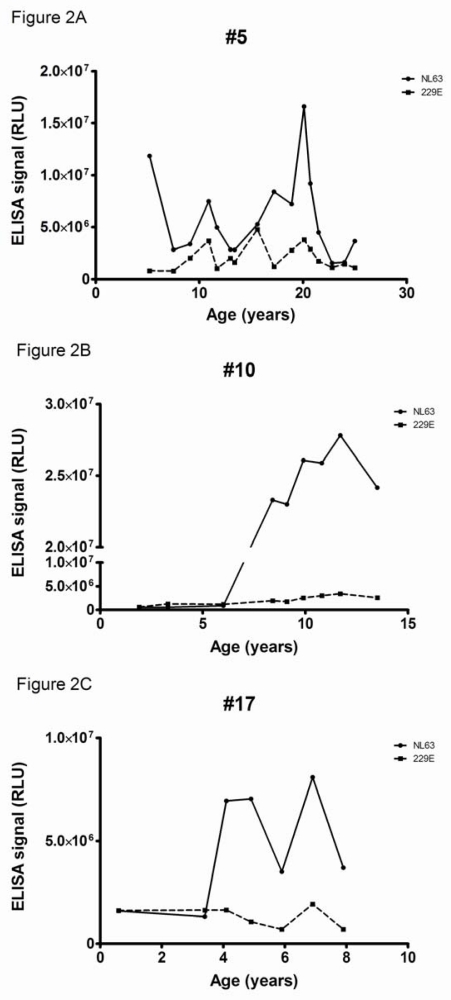
Serological response to HCoV-NL63 and HCoV-229E in time. Reactivity to the C-terminal nucleocapsid protein of HCoV-NL63 is shown as closed circles (continuous line), to the HCoV-229E C-terminal nucleocapsid protein closed squares (dashed line). (a) Rhesus Macaque 5; (b) Rhesus Macaque 10; (c) Rhesus Macaque 17. The X-axis displays the age in years of the Rhesus Macaque during follow up, the Y-axis the relative luminescence signal (RLU) in the two C-terminal nucleocapsid protein antibody ELISAs.

Longitudinal serum samples were available of three rhesus macaques with the highest antibody levels to HCoV-NL63 (numbers 5, 10 and 17). These monkeys had been sampled on a regular basis, with on average one serum sample every one or two years. Sampling started when the macaques were already a few years old, except for one which was followed from 6 months of age. In all three animals a sudden rise in antibody titers was noticed (Figure 2, panel A, B and C). Rhesus macaque number 5 was followed from 1989 (starting at the age of 6 years) with high levels of NL63-directed antibodies. A strong peak was observed in 2003, indicating that the animal experienced an additional HCoV-NL63 infection (at 20 years of age). Animal number 10 started with low titers to HCoV-NL63 (age 2 years) but after some years very high titers to HCoV-NL63 were visible (age 8 years). Unexpectedly, these high antibody levels remained for the complete follow-up period (last sample collected at the age of 13). This is suggesting that the adaptive immune response to HCoV-NL63 is constantly activated, and one could speculate that this animal is experiencing a chronic HCoV-NL63 infection. Unfortunately, additional samples like throat swabs or faeces to test for chronic infections were not available. The third animal (number 17) was followed from early age on, starting with low levels of NL63-antibodies. Between the years 2003/2004 a steep rise in antibody levels was noted, whereas a second rise occurred after the winter of 2006/2007 (at 4 years and almost 7 years of age respectively). The animals were all checked for antibodies to HCoV-229E, but for none of the three monkeys reactivity was detected (Figure 2). This is in accordance with the first screening of the animals, which already showed low levels of HCoV-229E antibodies in these animals.

**Figure 3.**
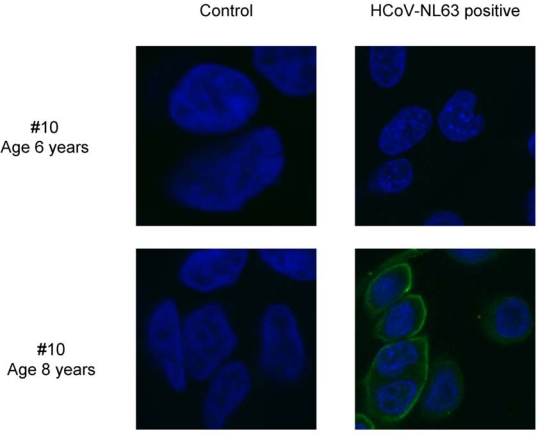
Immunofluorescence detection of HCoV-NL63 in infected LLC-MK2 cells. Reactivity of rhesus macaque number 10 sera towards HCoV-NL63 infected (HCoV-NL63 positive) and non-infected (control) LLC-MK2 cells, at the age of 6 (preseroconversion) and 8 (postseroconversion) years. The images represent the cell nucleus (blue) and rhesus macaque antibodies (green) signals.

To confirm our findings we determined whether the post-seroconversion samples also react with whole virus by performing an immunofluorescence assay with HCoV-NL63 infected cells. In all cases with low HCoV-NL63 antibodies we saw no reactivity with the infected cells. Whereas all samples obtained after seroconversion showed clear reactivity with whole virus (shown for animal number 10 in Figure 3).

With the assay used we cannot obtain evidence that it were HCoV-NL63 infections that these monkeys have encountered. However, one can conclude that it were infections with HCoV-NL63, or a virus which is serologically closely related. It can never be excluded that there is a coronavirus infecting these rhesus monkeys that is closely related to HCoV-NL63 (but has not been identified yet). Sampling of the respiratory tract on a regular basis throughout the years and eventually identify the pathogen that is eliciting the antibody response can answer this question, but is not an easy task. To our opinion the most likely explanation would be that natural infection with HCoV-NL63 occurs. The macaques described in this study live in a closed system with close contacts to humans (feeding, cleaning of cages, *etc*.). Through this contact they can encounter the virus as infections of humans with HCoV-NL63 occurs frequently in winter seasons [10]. As the cells of this monkey species are susceptible to infection (LLC-MK2 cells, a kidney epithelial cell line) it is very likely that *in vivo* infections can occur as well.

The animals of our study were clinically examined on a routine yearly basis for colony health surveillance, but subclinical infections with coronaviruses will go unnoticed during this check up and in the periods between the examinations. Thus it can not be determined whether the seroconversion or reconversion to HCoV-NL63 was accompanied with minor clinical signs. Future studies should be designed to investigate viral infection with human viruses and the accompanying clinical signs to further unravel this subject. Here we show that it is not necessary to sacrifice monkeys by experimental infecting. A well-designed follow up through the years with serum and respiratory samples collected on a regular basis would be sufficient to unravel the potential of the human respiratory viruses to infect rhesus macaques. Once a cohort has set up and samples have been collected for a few years, all newly identified respiratory viruses can be screened with the appropriate serological assays, and infection related to clinical symptoms.

Natural infections of rhesus macaques with viruses that are known to be of human origin are not unusual. It has been described that smallpox, measles, rubella or parainfluenzavirus 1 and 2 cause natural infection of Rhesus macaques and the closely related cynomolgus macaques [15,16]. Furthermore, experimental infection with several viruses showed that the animals are susceptible to various human respiratory viruses like respiratory syncytial virus and SARS coronavirus [17,18]. With the knowledge that natural infection of HCoV-NL63 is likely to occur, one should keep in mind that this has implications for animal model experiments. In case rhesus macaques are experimentally infected with HCoV-NL63 this might be a reinfection, in case the animal has encountered the virus previously, or the first infection, in case the animal is still young and was not naturally infected through human contact. The clinical signs during the first infection might be of a more severe nature in comparison with a recurrent infection. This should be taken into account in case a study is conducted to reveal the pathogenesis of the virus in an animal model system.

## 3. Experimental Section

### Serum of Rhesus Macaque monkeys

Cross-sectional and longitudinal serum specimens were collected from Rhesus macaques with an Indian, Burmese or Chinese genetic background. The Rhesus macaques were either imported or born in The Netherlands in one of the breeding colonies. The animals have not been isolated but remained in a breeding colony and were not used for immunization with antigens or adjuvant, or any other study. All serum specimens were stored at −80°C and heat-inactivated at 56°C for 30 minutes prior to analysis.

### Generation and expression of recombinant HCoV-NL63 and HCoV-229E carboxyl-terminal nucleocapsid proteins

The generation of the plasmid construct was performed as described [9]. For HCoV-NL63 the following primer combination was used 5′ NL63_N5_CT (5′ – CACCAAACCTAATAAGCCTCT TTCTCAAC – 3′) and 3′ NL63_Nexp (5′ – TTAATGCAAAACCTCGTTGAC – 3′), whereas for HCoV-229E the primer combination 5′ 229E_5N_CT (5′ – CACCCCTTCTCGTAATCAGAGTCCT – 3′) and 3′ 229E_Nexp (5′ – TTAGTTTACTTCATCAATTAT – 3′) was used. The generated pET100_NL63_CT and pET100_229E_CT plasmids were sequenced and shown to be 100% identical to the virus reference sequences of HCoV-NL63 (Amsterdam-01) and HCoV-229E (Inf-1), respectively. Subsequent expression and purification of the HCoV-NL63 and HCoV-229E recombinant carboxyl-terminal nucleocapsid proteins was performed as previously described [9].

### Carboxyl-terminal nucleocapsid ELISA

Ninety-six-well ELISA plates (Greiner Bio-one) were coated overnight at 4°C with 3 μg/ml of expressed recombinant C-terminal N protein of HCoV-NL63 or HCoV-229E. The proteins were diluted in 0.1 M carbonate buffer pH 9.6. Unspecific binding sites were blocked with PBS + 0.1% Tween20 (PBST) supplemented with 5% skim milk (Fluka) for one hour at room temperature (RT). Longitudinal and cross-sectional sera were diluted 1:200, in PBST containing 1% skim milk and incubated on the plate for 2 hours at RT. After washing, Alkaline Phosphatase conjugated anti-monkey IgG antibody (Sigma Aldrich) diluted (1:40,000) in 1% skim milk PBST was added. Following one hour at RT the plates were washed and signal was developed with 50 μl of Lumi-Phos Plus (Lumigen). Measurements were done with a Glomax™ 96 Plate Luminometer (Promega). All sera were tested in duplicate.

### Carboxyl-terminal nucleocapsid competition ELISA

Rhesus Macaque sera were diluted (1:200) in PBST containing 1% skim milk and twofold serial dilutions ranging from 50 to 0 μg/ml of either expressed recombinant C-terminal N protein of HCoV-NL63, N protein of HCoV-229E or LacZ protein and incubated for 1 hour at RT. Prior to incubation, the mixtures were briefly homogenized by vortexing. No centrifugation was performed. Following the pre-incubations the samples were measured by HCoV-NL63 or HCoV-229E ELISA, as described above.

### Immunofluorescence on HCoV-NL63 infected cells

LLC-MK2 cells were seeded on coverslips in 12-well plates at a density of 0.32 × 106 cells / well and cultured at 37 °C with 5% CO2 in minimal essential medium (MEM; 2 parts Hanks’ MEM and 1 part Earle’s MEM) supplemented with 3% heat-inactivated fetal calf serum (PAA Laboratories), penicillin (100 U/ml), and streptomycin (100 μg/ml). Cells were infected with HCoV-NL63 at a multiplicity of infection of 0.007 and incubated at 37 °C with 5% CO2. After 4 days cells were fixated with 3.7% PFA in PBS for 30 minutes at room temperature (RT). The unspecific binding sites were blocked overnight at 4 °C with incubation buffer (2 % BSA (Sigma Aldrich) in PBS with 0.1% saponin and 50 mM ammonium chloride). For detection of HCoV-NL63 proteins cells were double stained for 1 hour at RT in incubation buffer with rabbit derived anti-S1-NL63 sera (1:200) and Rhesus macaque sera (1:100) as primary antibodies. Donkey derived, Dylight 649 labeled, anti-rabbit IgG (H+L) and donkey derived, Dylight 594 labeled, anti-human IgG (H+L) (1:200) (Jackson immunoresearch) were applied as secondary antibodies for 1 hour at RT in incubation buffer, followed by nuclear DNA staining with Hoechst 33528. Fluorescent images were acquired on a Leica TCS SP2 AOBS spectral confocal microscope with a 63x HCX PL APO 1.32 oil objective.

## 4. Conclusions

In the early days of human coronavirus identification (the 1960s) it was possible to experimentally infect volunteers with the viruses which were isolated from persons with common colds. Nowadays, with the recent epidemic of SARS-CoV, it is very difficult to attract volunteers for such studies. Not only has the public become aware of the potential dangers of a coronavirus infection, the recently identified viruses (HCoV-NL63 and HCoV-HKU1) have been isolated from children or adults with severe lower respiratory tract infection, and not simple common colds. Consequently, the only possibility to connect the recently identified coronaviruses to a certain disease is via associations with disease or animal experiments. To date no animal model system has been described for HCoV-NL63. Although all signs suggest that Rhesus macaques might be a good model system – since cells of Rhesus macaques can be *in vitro* infected by HCoV-NL63 – nobody has reported infection of these animals yet. We show here that there are clear signs that Rhesus macaques acquire natural infections with HCoV-NL63, or a serologically very closely related coronavirus. In future animal experiments to be carried out with Rhesus macaques the history of an animal should be known to determine whether the experimental infection occurs in an HCoV-NL63 naïve animal or an animal that has encountered the virus during life, as clinical symptoms might differ greatly in first or recurrent infection.

## Figures and Tables

**Table 1.** Demographic characteristics of the Rhesus macaques included in HCoV-NL63 antibody analysis.

**Rhesus Macaque ID**	**Sampling date**	**Date of birth**	**Genetic origin**
1	17-4-2008	26-3-1983	India
2	24-4-2008	11-12-2000	India
3	29-9-2008	4-5-1982	India
4	29-9-2008	6-7-1983	India
5	29-9-2008	16-10-1983	India
6	6-10-2008	1-1-1996	Burma
7	6-10-2008	9-8-1996	Burma
8	6-10-2008	12-4-1989	Burma
9	20-10-2008	6-4-2001	India
10	27-10-2008	7-5-1995	Burma
11	15-8-2007	7-1-1995	China
12	15-8-2007	14-4-1995	China
13	17-4-2007	20-9-1994	China
14	17-4-2007	16-6-1994	China
15	3-4-2008	6-8-1980	India
16	10-4-2008	24-4-2000	India
17	10-4-2008	1-5-2000	India
18	10-4-2008	20-5-1995	Burma
19	14-4-2008	3-12-1988	Burma
20	14-4-2008	31-12-1988	Burma
21	27-10-2008	12-1-1997	Burma
22	27-10-2008	26-1-1997	Burma
23	12-1-2009	4-1-1996	India
24	12-1-2009	11-3-1996	India
25	5-1-2009	11-3-2000	India
26	29-12-2008	9-4-2001	India
27	29-12-2008	13-3-2000	India
28	13-1-2009	26-9-1995	Burma
29	24-8-2000	5-2-1995	China
30	30-1-2003	24-4-1995	China
31	8-5-2006	27-12-1994	China
32	4-2-2002	23-3-1995	China
